# High Fire Drives the Reorganization of Taiga Soil Fungal Communities with Ascomycota as the Dominant Phylum After Long-Term Recovery

**DOI:** 10.3390/jof11110772

**Published:** 2025-10-27

**Authors:** Siyu Jiang, Zhichao Cheng, Hong Pan, Siyuan Liu, Huijiao Qu, Mingliang Gao, Libin Yang, Jia Zhou

**Affiliations:** 1 School of Geographical Sciences, Harbin Normal University, Harbin 150025, China; 13363960039@163.com (S.J.); 15045728093@163.com (H.Q.); 2 Key Laboratory of Biodiversity, Institute of Natural Resources and Ecology, Heilongjiang Academy of Sciences, Harbin 150040, China; chengzc928@163.com (Z.C.); panhong500@163.com (H.P.); liuliu9826@163.com (S.L.); 3 Heilongjiang Huzhong National Nature Reserve, Huzhong 165038, China; zrbhjzhk@163.com

**Keywords:** soil fungal communities, boreal forests, wildfire disturbance, long-term post-fire recovery

## Abstract

Forest fires are key disturbance factors in forest ecosystems, and soil fungi play an irreplaceable role in post-fire recovery. This study focused on forest areas burned in 2000 in the Daxing’anling region of China, targeting long-term recovery sites with different fire intensities. Illumina MiSeq sequencing was used to analyze the structural characteristics of fungal communities and their environmental drivers. Results showed that compared with the control check (CK), the Shannon index of the low fire group (L) increased significantly (*p* < 0.05), while moderate (M) and high (H) fire groups reduced fungal diversity significantly. PCoA indicated significant differences in community structure (R^2^ = 0.97, *p* = 0.001). In highly burned areas, the relative abundance of Ascomycota reached 94.17%, and Basidiomycota lost its dominance. Spearman analysis showed that pH, available phosphorus, available potassium, soil fluorescein diacetate hydrolase, soil dehydrogenase, and soil urease were significantly positively correlated with fungal alpha diversity. RDA revealed that total nitrogen, available phosphorus, soil water content, alkaline nitrogen, active potassium, and dissolved organic carbon had extremely significant effects on soil fungal community composition (*p* < 0.01). Co-occurrence network analysis indicated that symbiotic relationships dominated all groups. Networks in L and M groups were more complex, while that in H group was simplified and severely damaged. This study indicated that after long-term recovery, soil fungal communities in low fire areas returned to pre-fire levels; those in moderate and high fire areas did not recover, with high fire burns causing severe damage and community structure reorganization.

## 1. Introduction

Fire, as the most common and important disturbance factor in forest ecosystems, has shown a significant increasing trend in its frequency and intensity against the backdrop of global climate change [1]. Forest fires trigger drastic changes in the forest environment, not only directly destroying surface vegetation but also indirectly affecting the structure and function of forest ecosystems by altering soil physical and chemical properties, enzyme activities, and soil biological activity [2]. The depth of impact depends on multiple factors such as fire intensity, duration, and the recovery capacity of the ecosystem. Furthermore, the effects of forest fires on ecosystems have dual attributes: ecological and destructive. On one hand, fires damage the existing ecosystem structure, causing devastating harm to surface vegetation and underground organisms; on the other hand, fire disturbances can promote vegetation succession and renewal, accelerate the optimization of soil nutrient cycling, enhance soil biological functional activity, and thereby drive the self-regulation and recovery of ecosystems [3].

Soil fungi, as a crucial component of soil microbial communities, exhibit high sensitivity to fire disturbances and rapid respond to changes in soil physical and chemical properties. They serve as key indicators for evaluating the ecological recovery process of burned forestlands and play an irreplaceable role in maintaining ecosystem functions [4,5]. Currently, research on the recovery of soil fungal communities after forest fire disturbances remains controversial. For example, Kutorga et al. [6] demonstrated that the relative abundance of soil fungi was able to return to the level observed in unburned areas within two years following a fire. Cheng et al. [7] observed that in the middle stage of post-fire recovery, community composition changed significantly: the relative abundance of Basidiomycota decreased while that of Ascomycota increased, which was hypothesized to be related to the lignin-degrading ability of Basidiomycota. However, Sun et al. [8] and Duhamel et al. [9] in studies on a longer time scale, found that Ascomycota was gradually replaced by Basidiomycota, and the abundance of ectomycorrhizal fungi recovered. They further pointed out that soil pH and carbon content were identified as the dominant factors driving these dynamics. In addition, forest fire significantly alters soil environments (such as temperature, humidity, and pH) and nutrient supply, thereby affecting the succession of fungal communities in burned forestlands [10]. For instance, high temperatures generated by fires can directly kill fungi in the soil surface layer, while residual ash releases alkaline ions, increasing soil pH [11]. Changes in pH may cause physiological stress to certain fungal taxa, leading to the loss of their competitive advantages, with saprophytic fungi being most significantly affected [12]. Dominant genera in Basidiomycota are mostly ectomycorrhizal fungi, which can more efficiently acquire nutrients by forming symbiotic relationships with plant roots. As the soil environment returns to a state suitable for reproduction over post-fire recovery time, they gradually dominate during the recovery process [13]. In summary, through directly killing soil fungi and indirectly altering the soil environment, forest fires not only reshape fungal community structure but also may inhibit the colonization or diversity of key functional taxa, weakening the response capacity of fungal communities and thereby delaying the recovery rate of post-fire forest ecosystems. It is worth noting that forest fire has significant long-term effects on soil fungi, and there are obvious differences in the time required for their recovery to pre-fire levels, mainly depending on the synergistic effects of multiple factors such as fire intensity, post-fire climatic conditions, and soil environmental factors [14].

The Daxing’anling forest area is one of the regions with frequent cold-temperate forest fires in China. The regional forest community is dominated by *Larix gmelinii*, which exhibits high sensitivity to climate change [15]. Currently, research on forest recovery in burned areas of the Daxing’anling mainly focuses on the short-term acute responses of microorganisms after fires, or on aboveground vegetation reconstruction and changes in soil physical and chemical properties following long-term recovery [16]. However, as an important component of soil microorganisms, research on the long-term recovery of soil fungi after fires in this region remains relatively scarce. This study focuses on long-term post-fire recovery plots in cold-temperate larch forests as the research object. Using Illumina MiSeq high-throughput sequencing technology (Illumina, San Diego, CA, USA) to analyze the community structure and diversity of soil fungi under different fire intensities, aiming to explore the environmental factors influencing changes in soil fungal communities during the long-term post-fire recovery process and investigate the interaction patterns of soil fungal communities in the post-fire context. This research holds important theoretical and practical significance for understanding ecosystem recovery processes under fire disturbances and for adaptive forest management.

## 2. Materials and Methods

### 2.1. Study Area

The research site is situated within Huzhong National Nature Reserve, Heilongjiang Province, China, with geographical coordinates spanning from 122°42′14″ to 123°18′05″ E and 51°17′42″ to 51°56′31″ N. The region exhibits a cold-temperate continental monsoon climate, characterized by long, severe winters with heavy snowfall and short, warm summers with abundant rainfall. Annual mean temperature in this area is −4 °C, with average annual precipitation and evaporation reaching 458.3 mm and 911 mm respectively. At present, Huzhong National Nature Reserve ranks among China’s most characteristic and well-preserved cold-temperate coniferous forest ecosystems [17].

### 2.2. Sample Plots and Sample Collection

Since the wildfire occurred in the study area, it has been in a long-term recovery stage, and the forest stand structure and community composition have not yet fully returned to the pre-fire level. With the gradual recovery of forest stands, the understory environment has been constantly changing, and the overall shrub diversity has shown an upward trend. The dominant shrub species are mainly *Rhododendron tomentosum* Harmaja, *Vaccinium vitis-idaea* L., and *Betula fruticosa* Pall. The arbor layer is mainly composed of *Betula platyphylla* Suk. And *Populus davidiana* Dode, while the herb layer is dominated by *Deyeuxia korotkyi* (Litv.) S. M. Phillips & W. L. Chen, *Carex caespitosa* L., and *Saussurea japonica* (Thunb.) DC. Against this backdrop, to explore the long-term recovery of soil fungal communities after forest fire disturbance, forestlands in the reserve that experienced a fire in 2000 were selected as the long-term recovery study area (Table 1) [18]. In July 2024, according to the fire severity classification standard (Table 2) [19], experimental plots with low (L), moderate (M), and high (H) fire intensity were established within the study area. Additionally, control check (CK) were selected in adjacent non-burned areas similar to the fire-affected sites in terms of elevation, vegetation composition, and environmental conditions. A total of 28 plots were established, each measuring 20 m × 20 m, with seven replicates for each treatment. The dominant soil type in the study area is brown coniferous forest soil (WRB: Cambisols), which is a typical zonal soil formed under boreal coniferous forests. After removing the litter and humus layers, soil samples were collected from the 0–20 cm depth. All samples were transported to the laboratory in insulated containers at −20 °C. The soil was then passed through a 2 mm sieve to remove gravel, plant roots, and other debris. After processing, soil samples were divided into two parts. One part was employed for measuring soil physicochemical properties and enzyme activities as previously described [20], and the other part was used for analyzing fungal diversity.

### 2.3. Extraction and Sequencing of Fungi DNA

Total DNA was extracted from soil samples using the E.Z.N.A.^®^ Soil DNA Kit (Omega Bio-tek, Norcross, GA, USA) and diluted to 1 ng/μL after electrophoresis detection. The fungal rRNA gene ITS region was amplified using the primers ITS1F (5′-CTTGGTCATTTAGAGGAAGTAA-3′) and ITS2R (5′-GCTGCGTTCTTCATCGATGC-3′). The amplification protocol was as follows: pre-denaturation at 98 °C for 1 min, 30 cycles including (98 °C for 10 s, 50 °C for 30 s, and 72 °C for 30 s); and a final extension at 72 °C for 5 min. Amplification was performed using a PCR instrument (Bio-rad T100 Gradient PCR Instrument Bio-Rad Laboratories, Hercules, CA, USA). PCR products were mixed in equal volumes according to their respective concentrations. After thorough mixing, the products were purified by electrophoresis on a 2% agarose gel prepared with 1 × TAE buffer. Following purification with the QIAquick PCR Purification Kit (Qiagen, Hilden, Germany), library quality was inspected using the Agilent 5400 System (Agilent Technologies, Santa Clara, CA, USA). Qualified libraries were subsequently sequenced on the Illumina MiSeq platform (Illumina, San Diego, CA, USA). The raw data were uploaded to the NCBI SRA database (sequence number: PRJNA1162305).

Raw sequencing data were subjected to quality control using fastp software [21] (https://github.com/OpenGene/fastp, version 0.20.0, accessed on 6 September 2024), and subsequent sequence merging was conducted with FLASH software [22] (e (http://ccb.jhu.edu/software/FLASH; v1.2.7, accessed on 6 September 2024). ASVs were obtained by denoising using the DADA2 [23] plugin in the QIIME 2 (version2023.9). Based on unite8.0/its_fungi (http://unite.ut.ee/, v8.0) species annotation database, we used the Naive Bayes classifier in Qiime2 for species taxonomic analysis of ASVs. Subsequent data analysis was conducted through the Microbial Cloud Diversity Analysis Platform (QIIME 2 process) provided by Shenzhen Microcommon Technology Group Co., Ltd. (https://www.bioincloud.tech/, accessed on 10 September 2024).

### 2.4. Data Analysis

Following sequencing processing of the original data, data collation and statistical analysis were performed using Origin 2021, Excel 2021, and SPSS 22.0 software. One-way analysis of variance (ANOVA) was conducted on soil physicochemical properties, enzyme activities, fungal community diversity indices, and community composition, and Duncan’s test was used to compare differences among groups. The Chao 1 index and Shannon index were used to characterize the alpha diversity of the fungal community. Principal Coordinates Analysis (PCoA) based on Bray–Curtis distances was performed, and permutational multivariate analysis of variance (PERMANOVA) was combined to test the significance of inter-group differences, thereby evaluating the beta diversity of fungal communities among samples. Redundancy analysis (RDA) and Spearman correlation analysis were used to elucidate the associations between the fungal community and relevant environmental factors. The co-occurrence networks of the fungal community were constructed using the “igraph” and “psych” packages of the R-3.3.1. Network visualization was implemented with Gephi.10.1 software, and network topological parameters were calculated to evaluate the complexity and stability of the community.

## 3. Results

### 3.1. Changes in Soil Physicochemical Properties and Enzyme Activities During Long-Term Recovery of Burned Forests

Soil physicochemical characteristics and enzyme activities of the chosen plots with low, moderate, high, or no fire disturbance have been previously described [20] and are briefly summarized as follows: Compared with CK, the contents of SOC, BC, TN, AN, AP, and AK in the soil increased significantly after fire (*p* < 0.05), while the WC content decreased significantly. In the fire groups, the AK content increased significantly with fire intensity, and the pH exhibited an initial increase followed by a decrease; the L and M groups showed significantly higher soil pH than that of CK, while H was significantly lower than that of CK. Compared with CK, the soil MBC and DOC contents increased significantly in L and H groups but decreased significantly in M. S-SC activity was significantly lower than that in the CK group (*p* < 0.05), whereas S-UE, FDA, and S-DHA activities were significantly higher than those in CK. Notably, S-UE activity increased as fire intensity rose. Among all groups, the activities of S-UE, FDA, S-ACPT, and S-DHA in the H were significantly higher than those in the other groups.

### 3.2. Recovery Characteristics of Soil Fungal Community Diversity

**Figure 1:**
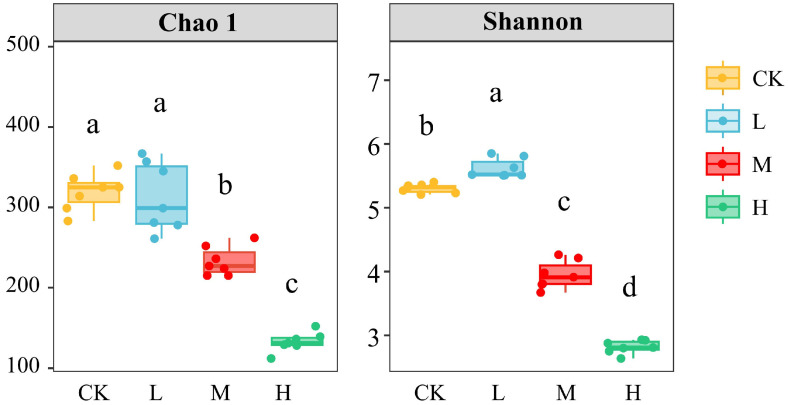
Alpha diversity of fungal communities in soil. Values are expressed as mean ± standard error (*n* = 7). Different letters in the same row indicate statistically significant differences (*p* < 0.05, ANOVA) across the various fire intensity levels in this study.

As shown in Figure 1, compared with CK, the Chao 1 index in L showed no significant change, while the Shannon index increased significantly (*p* < 0.05). The Chao 1 and Shannon indices in the M and H groups decreased significantly with increasing fire intensity. The results indicate that after long-term recovery, there were significant differences in the diversity and richness of soil fungal communities in burned forest areas, with only the alpha diversity of soil fungi in L recovered to the pre-fire level.

**Figure 2:**
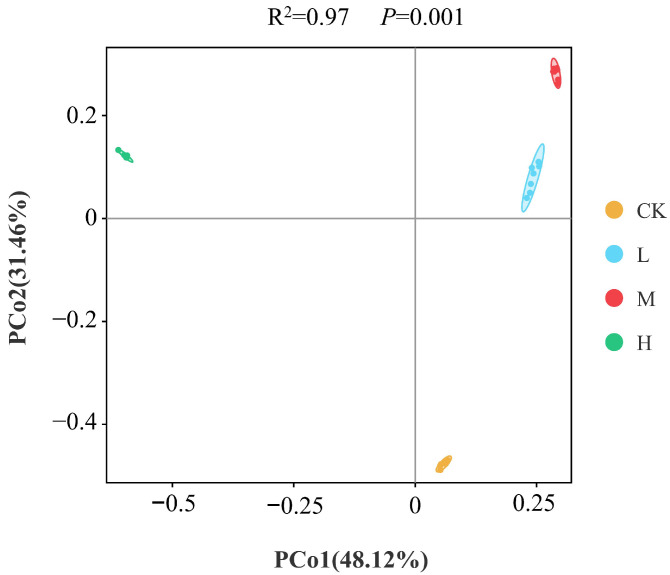
Principal coordinate analysis (PCoA) of soil fungal communities. Values are expressed as mean ± standard error (*n* = 7). Different colored dots represent control (CK, no fire), low fire (L), moderate fire (M), and high fire (H).

Fungal community beta diversity was analyzed based on Bray–Curtis distance using principal coordinate analysis (PCoA). As shown in Figure 2, the explanatory rates of PCo1 and PCo2 axes were 48.12% and 31.46%, with a cumulative interpretation of 79.58%. As shown in Table 3, PERMANOVA test revealed significant differences in soil fungal community structure between fire groups and the CK group (R^2^ = 0.97, *p* = 0.001). Specifically, the CK group clustered in the fourth quadrant, while the L and M groups were distributed in the first quadrant, and the H group in the second quadrant. The research results indicate that there are significant differences in soil fungal community structures in cold-temperate forests under different intensity disturbances, and the beta diversity of fungal communities has been significantly changed after long-term recovery.

### 3.3. Recovery Characteristics of Fungal Community Composition and Abundance

**Figure 3:**
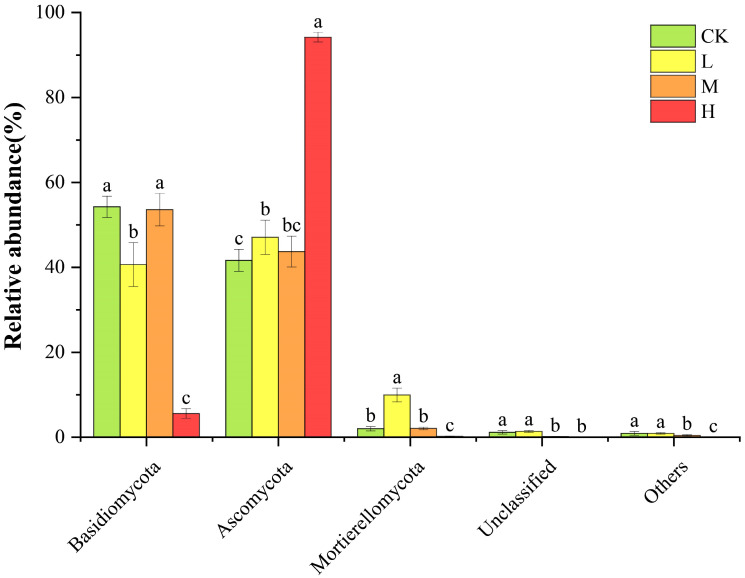
Soil fungal community composition at the phylum level. Different letters above bars indicate significant differences (*p* < 0.05, ANOVA) among the different intensities of fire in this study.

As displayed in Figure 3, the dominant phylum of soil fungal communities was Ascomycota (41.66% to 94.17%). Compared with CK, its relative abundance in the H group increased significantly (*p* < 0.05). Basidiomycota (5.59% to 54.25%) remained a dominant phylum in the L and M groups. Compared with CK, its relative abundance in L decreased significantly (*p* < 0.05), showed no significant difference in M, and decreased significantly to the lowest level in H. The relative abundance of Mortierellomycota (0.20% to 9.95%) increased significantly in L (*p* < 0.05), showed no significant difference in M, and decreased significantly in H. The relative abundances of Unclassified and Others showed no significant difference in L but decreased significantly in both the M and H groups (*p* < 0.05). The results indicated that the soil fungal community composition exhibited significant differences after long-term post-fire recovery, with the relative abundances of Basidiomycota and Ascomycota undergoing significant changes; among them, the changes in H were the most significant.

**Figure 4:**
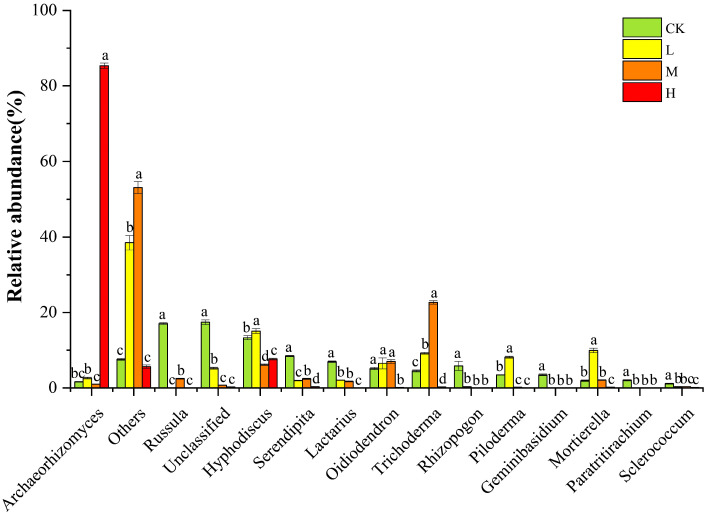
Soil fungal community composition at the genus level. Different letters above bars indicate significant differences (*p* < 0.05, ANOVA) among the different intensities of fire in this study.

As shown in Figure 4, the relative abundance of Archaeorhizomyces (0.98% to 85.33%) increased significantly in the H group (*p* < 0.05), becoming the dominant genus. The relative abundances of Others (5.65% to 53.09%) and Trichoderma (0.26% to 22.70%) increased significantly in the L and M groups. Compared with CK, the relative abundances of Russula, Unclassified, Serendipita, Lactarius, Rhizopogon, Geminibasidium, Paratritirachium, and Sclerococcum decreased significantly in the fire groups. These results indicated that the soil fungal community composition was markedly altered after long-term post-fire recovery, with Archaeorhizomyces (Ascomycota) showing a significant increase in the H group.

### 3.4. Correlation Between Fungal Community Structure and Soil Environmental Factors

#### 3.4.1. Correlation Analysis of Fungal Species, Diversity Indices and Soil Environmental Factors

**Figure 5:**
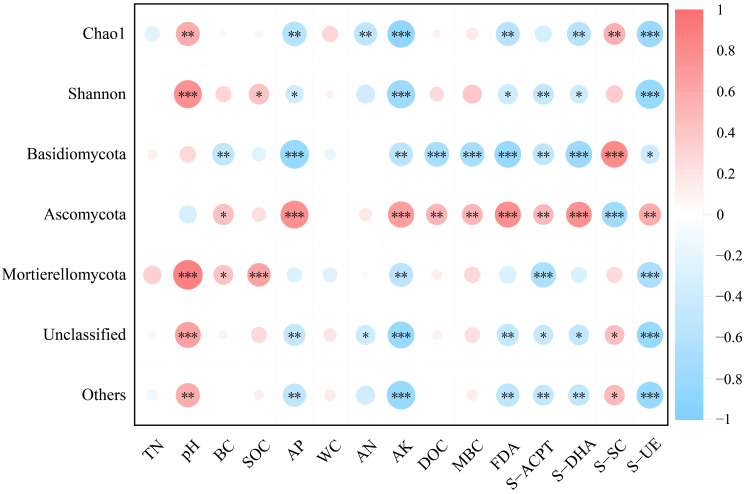
Heatmap of correlations between soil fungal community diversity, soil physicochemical properties, and enzyme activities. Positive correlations are marked in red and negative correlations in blue, where deeper colors correspond to stronger correlation strength. Significance is denoted as follows: * *p* < 0.05, ** *p* < 0.01, *** *p* < 0.001.

As illustrated in Figure 5, the Chao 1 index exhibited an extremely significant positive correlation with pH (*p* < 0.01), while showing extremely significant negative correlations with AP, AN, and AK. The Shannon index was extremely significantly positively correlated with pH, significantly positively correlated with SOC (*p* < 0.05), significantly negatively correlated with AP and AK. pH, AP, and AK are the main factors affecting soil fungal alpha diversity. The Chao 1 index was extremely significantly negatively correlated with FDA, S-DHA, and S-UE (*p* < 0.01), and extremely significantly positively correlated with S-SC. The Shannon index was extremely significantly negatively correlated with S-ACPT and S-UE, and significantly negatively correlated with FDA and S-DHA.

Correlation analysis was performed between soil fungal species at the phylum level and soil physicochemical properties as well as enzyme activities. As illustrated in Figure 5, BC, AP, AK, DOC, and MBC all showed significant negative correlations with Basidiomycota (*p* < 0.05), yet significant positive correlations with Ascomycota. In contrast, pH, BC, and SOC were significantly positively correlated with Mortierellomycota, while AK exhibited a significant negative correlation with this phylum. Among these, AP, AK, and pH are the main factors affecting the distribution of fungal phyla. FDA, S-ACPT, S-DHA, and S-UE were significantly negatively correlated with Basidiomycota (*p* < 0.05) but significantly positively correlated with Ascomycota. S-SC was extremely significantly positively correlated with Basidiomycota (*p* < 0.01) but extremely significantly negatively correlated with Ascomycota. In addition, S-ACPT and S-UE were extremely significantly negatively correlated with Mortierellomycota.

#### 3.4.2. Correlation Analysis Between Fungal Communities and Soil Environmental Factors Under Different Fire Intensities

**Figure 6:**
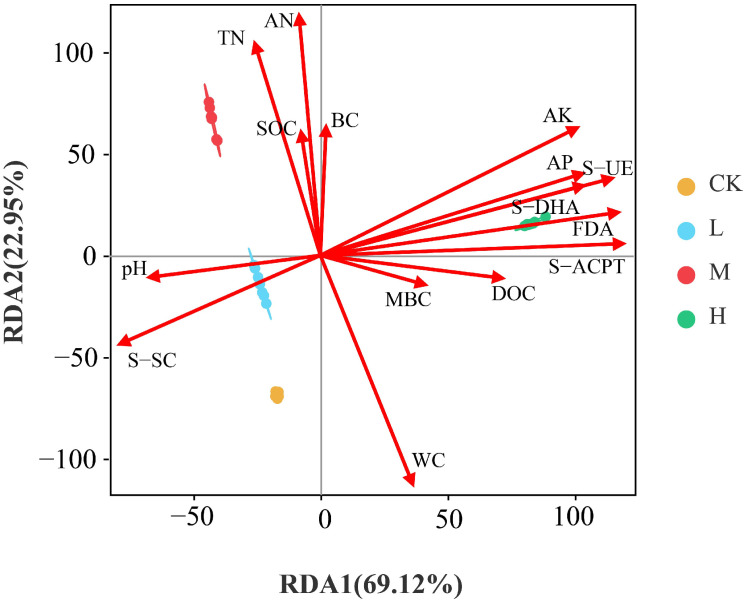
RDA of soil fungal community structures and soil physicochemical properties and enzyme activities. Red arrows indicate environmental factors.

The results of redundancy analysis (RDA) are shown in Figure 6. The explanatory rates of the RDA1 and RDA2 axes were 69.12% and 22.95% respectively, with a total explanatory rate of 92.07% for the two axes. The fungal community in L was positively correlated with pH and S-SC; M was positively correlated with TN, SOC, AN, and BC; H was positively correlated with TN, AN, SOC, BC, AK, AP, S-UE, S-DHA, FDA, S-ACPT, DOC, and MBC, and negatively correlated with pH, S-SC, and WC. As shown in Table 4, soil TN, AP, WC, AN, AK, and DOC had an extremely significant impact on soil fungal community composition (*p* < 0.01), while pH, BC, and SOC had a significant impact (*p* < 0.05). There was an extremely significant correlation between the activities of soil enzymes (FDA, S-ACPT, S-DHA, S-SC, and S-UE) and fungal community structure (*p* < 0.01).

### 3.5. Co-Occurrence Network Analysis of Soil Fungal Communities After Long-Term Recovery in Burned Forest

**Figure 7:**
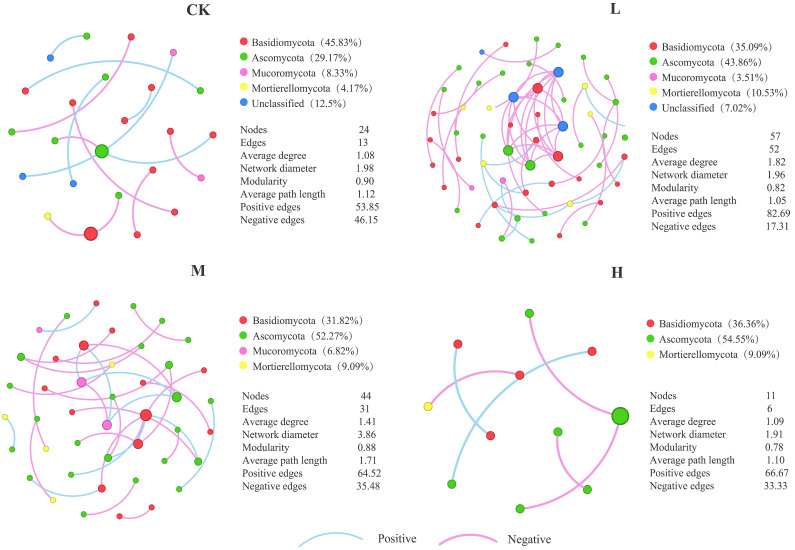
Co-occurrence network analysis and topological characteristics of soil fungal communities. Significant correlations (Spearman’s r > 0.6, *p* < 0.05) exist between taxa. The size of nodes corresponds to the degree of connectivity, and colors distinguish different phyla. Pink edges represent positive correlations, while blue edges represent negative correlations. Network nodes correspond to the relative abundance of each phylum. Topological parameters such as average path length, clustering coefficient, and modularity.

As presented in Figure 7, the soil fungal co-occurrence network analysis clarified the dynamics and interaction patterns of key fungal phyla during the long-term recovery process following fire disturbance. Among all nodes in the co-occurrence networks, Basidiomycota (31.82% to 45.83%), Ascomycota (29.17% to 54.55%), and Mortierellomycota (4.17% to 10.53%) were the dominant phyla in the co-occurrence networks of burned forestlands. Compared with the CK, the relative abundance of Basidiomycota in the fire groups decreased significantly, while that of Ascomycota increased significantly. Notably, Mortierellomycota became a dominant phylum in L (10.53%), with an abundance significantly higher than that in other groups, indicating that this phylum may have unique adaptability to low fire disturbance. In addition, differences in the abundance of non-dominant phylum nodes among groups also suggested that low-abundance taxa play an important role in maintaining soil fungal community assembly. The number of nodes, edges, and average degree in the L and M groups were all higher than those in CK, and the network diameter of M was larger than that of CK. These results indicated that long-term post-fire recovery increased the number of species involved in interactions and the intensity of interaction relationships, enhancing the complexity of soil fungal networks. Notably, L had the highest number of nodes, edges, average degree, and average clustering coefficient, as well as the lowest modularity value, suggesting that after long-term recovery, L contained the most abundant key species and the most intensive interaction relationships. Specifically, all topological parameters of H were lower than those of other groups, indicating that high fire has caused persistent and severe damage to soil fungal interaction networks, greatly reducing the number of key species and the complexity of interactions. Even after long-term recovery, its network structure and function failed to recover to the unburned level. In addition, in all groups, the proportion of positive correlations between nodes in the fungal co-occurrence networks was higher than that of negative correlations, indicating that the interaction relationships among soil fungal communities after fire recovery were more dependent on symbiosis rather than competition. Among them, the proportion of positive correlation edges in L (82.69%) was much higher than in other groups, suggesting that in the complex network of the low fire group after long-term recovery, key species formed a strong symbiosis-dominated interaction pattern.

## 4. Discussion

### 4.1. Effects of Long-Term Post-Fire Recovery on Soil Fungal Diversity

Microbial diversity indices are key indicators that characterize the response and adaptation capabilities of microbial communities to habitat changes, reflecting the recovery status and stability of soil ecosystems. In this study, it was found that after long-term post-fire recovery, both the Chao 1 richness index and Shannon diversity index of fungal communities in moderate and high fire groups were significantly reduced, with only the soil fungal alpha diversity in the low fire group recovering to the level of unburned areas. This result is consistent with previous research findings [13,24]. On one hand, due to the low heat resistance of soil fungi, fires have caused severe thermal damage to them, leading to the direct death of a large number of fungi [25]. On the other hand, the reduction in organic matter resources and significant changes in soil physical and chemical properties have further compressed the living space of fungi. Even after long-term recovery, fungal communities can only be dominated by some highly tolerant taxa, making it difficult to rebuild the original diversity [26]. The damage caused by low fires is relatively minor, and some tolerant species can recover quickly. Previous studies have found that moderate fire disturbances may be beneficial to the growth of soil fungi, and soil fungal alpha diversity can recover to the unburned level after long-term recovery [24].

In this study, the soil pH values of all burned groups was in an acidic environment, with the pH value of the high fire group showing a significant decrease. This environment not only screened out a few acid-tolerant fungal taxa to survive but also exerted stress on most fungal taxa, directly inhibiting their growth and activity, thereby reducing fungal community diversity [27]. It is worth noting that the activities of key enzymes such as FDA, S-DHA, and S-UE in the soil of burned groups were significantly enhanced, which is consistent with previous research results [28]. Among them, the enhancement of S-DHA enzyme activity reflects the improved metabolic activity of fungal taxa, enabling some dominant taxa to gain a competitive advantage by efficiently oxidizing refractory carbon sources (such as aromatic compounds). This intense resource competition significantly inhibits the growth of other fungal taxa, thereby reducing fungal community diversity [29]. Fungal taxa in moderate and high fire groups significantly enhanced S-UE enzyme activity to efficiently seize easily degradable nitrogen sources (quickly decomposing urea to obtain available nitrogen), forming resource monopolies to gain competitive advantages, which leads to the competitive exclusion of other taxa that rely on easily degradable resources [30]. Therefore, the synergistic effect of soil enzyme activities has reshaped the resource utilization patterns and competitive relationships of fungal communities, promoting the succession of communities toward dominance by specific functional taxa, and ultimately resulting in a decrease in community diversity.

### 4.2. Effects of Forest Fire on Soil Fungal Community Composition After Long-Term Recovery

Soil fungal communities are highly sensitive to changes in soil environmental factors. After high fire disturbance, various physical and chemical properties of the soil and the microenvironment of the forest undergo changes. During the long-term recovery process of burned forestlands, these changes affect the survival of soil fungi and play a key role in shaping community structure [31]. In this study, it was found that Ascomycota and Basidiomycota remained the dominant phyla in the low and moderate fire groups, which is consistent with previous research results [7,32]. This may be related to the ability of Ascomycota to degrade recalcitrant organic substances such as cellulose and lignin. During the long-term post-fire recovery process, recalcitrant lignin is gradually consumed, while new litter rich in cellulose and hemicellulose continuously accumulates. The significant change in litter composition favors the growth and reproduction of Ascomycota, which are adept at decomposing recalcitrant materials [32]. Redundancy analysis revealed that the soil fungal community structure in high fire group was significantly positively correlated with soil enzyme activities and contents of AP and AK. In high fire forestlands, the average relative abundance of Ascomycota significantly increased to 94.17%, and correlation heatmap analysis showed that its abundance was also significantly positively correlated with AP, AK, and other factors. This indicates that under conditions of significantly increased soil AP and AK contents, Ascomycota, which adopt an eutrophic ecological strategy, exhibit significantly enhanced ecological competitive advantages due to their ability to rapidly absorb nutrients and grow, thereby occupying dominant ecological niches and becoming the dominant taxa in the fungal community [33]. Archaeorhizomyces belongs to the phylum Ascomycota, and is typically distributed in the rhizosphere of plants, possessing both saprophytic and potential symbiotic characteristics [34]. In this study, the results of genus-level analysis showed that Archaeorhizomyces has become the dominant species in the highly burned and restored forestlands, and it exhibits a significantly positive correlation with soil AP content (Figure S1). A study by Chen Et Al. revealed that Archaeorhizomyces can sustain its growth by relying on root exudates and available organic matter in the soil, and its metabolic activities are highly sensitive to phosphorus availability [35]. Therefore, after the restoration of severely burned forestlands, the soil AP content increases significantly, providing a sufficient nutrient source for Archaeorhizomyces. Meanwhile, the damage to the fungal community releases part of the ecological niche space, which promotes the expansion of Archaeorhizomyces in the community and enables it to gain a competitive advantage, thus making it an important group in the high burned forestlands.

The increased abundance of Ascomycota further intensifies nutrient competition, limiting Basidiomycota’s access to resources, resulting in a significant decrease in the relative abundance of Basidiomycota in high fire group and the loss of their dominant position in the community. This may be related to the oligotrophic characteristics of Basidiomycota. Although they possess strong lignin-degrading capabilities [36], they often form symbiotic relationships with plant roots. The death or damage of trees and their roots due to fire disrupts this symbiosis. Even after long-term recovery, vegetation growth and soil conditions have not returned to levels suitable for their reproduction, making it difficult for them to obtain the carbon sources necessary for survival, thereby leading to a significant reduction in their relative abundance [7,13]. Additionally, in this study, the activity of S-SC was significantly reduced, indicating a decrease in simple sugars such as glucose and fructose hydrolyzed by sucrase. The insufficient supply of easily utilizable carbon sources and energy required for the growth and reproduction of soil fungi may have inhibited the growth of Basidiomycota [37].

During the long-term recovery process after fire disturbance, soil nutrient conditions are key driving factors shaping the structure of forest soil fungal communities and the succession of dominant taxa. After long-term recovery, the relative abundance of Mortierellomycota in low fire group was significantly higher than that in the control group, and its abundance showed an extremely significant positive correlation with soil pH and SOC content. As mentioned earlier, pH has a significant impact on saprophytic fungi, and as a typical r-strategist saprophytic fungus, soil pH is one of the important factors affecting the community changes of Mortierellomycota [38]. Meanwhile, this group of fungi also has the ability to degrade lignin, cellulose, and hemicellulose. After long-term recovery from low fire, the composition and quantity of the litter have changed, providing abundant nutrient sources for Mortierellomycota and promoting their rapid growth. Furthermore, as beneficial soil fungi, Mortierellomycota can stimulate plant growth and indirectly improve the rhizosphere soil environment [39], their increased abundance may further accelerate the process of post-fire vegetation recovery.

### 4.3. Co-Occurrence Network Analysis of Fungal Communities After Long-Term Recovery

Microbial network analysis can effectively resolve the interaction relationships of microbial communities under environmental changes. The complexity of network structure and topological properties of communities under different fire intensities can reflect the spatial distribution patterns of microorganisms and species coexistence relationships [40]. In this study, Basidiomycota and Ascomycota were the dominant phyla in the fungal networks of all groups. Network analysis showed a decreasing trend in the abundance of Basidiomycota, which was related to the strong environmental adaptability of Ascomycota. This altered the resource competition pattern within the community and weakened the niche advantage of Basidiomycota. Mortierellomycota became a key dominant group in the low fire group, which was closely associated with its efficient carbon source utilization capacity and strong stress tolerance; meanwhile, the soil microenvironment formed after long-term recovery provided suitable conditions for its growth. Overall, fungal communities regulated their community structure and function for stress resistance through interactions with environmental factors, maintaining community stability to a certain extent [41].

This study found that the topological parameters (e.g., number of nodes, number of edges, and average degree) of fungal co-occurrence networks in the low and moderate fire groups were significantly higher than those in the control group. This indicates that after long-term recovery, the interaction intensity among soil fungal communities was significantly enhanced, and the co-occurrence networks showed higher connection complexity, implying improved efficiency of soil nutrient cycling and energy flow [42]. This may be related to the redistribution of soil nutrients after low and moderate fires, which broke the original microbial competition pattern and promoted new microbial interactions, thereby increasing network complexity [43]. Notably, the topological parameters of the high fire group were significantly lower than those of the control group, possibly because high fires significantly altered soil physicochemical properties through direct thermal effects. Despite more than 20 years of recovery, the high fire group still showed significant inhibitory characteristics, with only a few stress-tolerant species remaining. The reduction in the number of key species and simplification of community structure led to a significant weakening of the complexity of interspecific interaction networks in soil microbial communities [44]. Interactions were concentrated in a few microbial groups, reflecting a decline in species diversity and the fragmentation of interaction networks, which is consistent with the previously mentioned significant decrease in fungal alpha diversity in the severely burned groups. Regarding interaction relationships within fungal communities, the proportion of positive correlations in co-occurrence networks was significantly greater than that of negative correlations—this indicates that symbiosis, rather than competition, was the dominant form of intercommunity interactions [45]. Specifically, this trend was most significant in the low fire group. This may be because low fires caused relatively minor damage to the soil environment, not only retaining most organic matter and niche diversity but also forming specific environmental conditions that provided abundant resources for microorganisms, thereby promoting the formation of symbiosis relationships [46]. These results suggest that moderate fire disturbances can promote the network reconstruction of fungal communities. In contrast, the unburned forestlands had a higher proportion of negative correlations, indicating more significant competitive relationships in their fungal communities. Species in the unburned groups exhibited a significant reduction in niche width due to community differentiation, which further intensified interspecific resource competition, consistent with previous research findings [47].

After long-term recovery of burned forestlands, microbial communities formed a balanced interaction pattern dominated by symbiosis and supplemented by competition. Soil fungal communities constructed more complex interaction networks than unburned forestlands. The complexity of fungal co-occurrence networks built after long-term recovery was significantly higher than that of unburned forest lands, reflected in enhanced topological structure and improved stability of key community functions, ultimately forming a soil microbial community ecosystem with stronger disturbance resistance.

## 5. Conclusions

In the long-term recovery stage following forest fires, the Alpha diversity of soil fungal communities in the Daxing’anling taiga forests has recovered to the pre-fire level in low fire forestlands, but the Beta diversity remains significantly different. In high fire recovery forestlands, Ascomycota has replaced Basidiomycota to gain a competitive advantage, and Archaeorhizomyces is the dominant species in this group. Factors such as AP and AK are important drivers of changes in soil fungal community structure. The soil fungal communities in all burned forestlands are dominated by symbiotic relationships, but there are differences in network complexity; the complexity of fungal community networks in low and moderate fire forestlands has increased, while the network structure in high fire forestlands has been simplified.

## Figures and Tables

**Table 1:** Successional recovery stages of post-fire boreal forests.

Recovery Stage	Time Scale	Character
Short Term	0–7 years	Dominated by light-demanding herbs and shrubs
Medium Term	7–16 years	Dominated by shade-tolerant herbs
Long Term	16–30 years	Dominated by birch as the primary tree species, with shade-tolerant herbs and shrubs in the understory

**Table 2:** Classification standards for varying fire intensities.

Intensity	Flame Altitude	Standing Timber Harm	Character
Low Fire (L)	≤1.5 m	≤30%	Bark and trunk scorched, but crown retains green foliage
Moderate Fire (M)	1.5–3 m	30–70%	Trunk charred, but partial green foliage remains
High Fire (H)	≥3 m	≥70%	Crown consumed, no green foliage retained

**Table 3:** Multivariate test analysis based on Bray–Curtis distance. Significance is denoted as follows: * *p* < 0.05, **p < 0.01, *** *p* < 0.001.

Group	R^2^	*p*-Value	*p*-adj.BH
CK vs. L	0.918	0.001	0.002 **
CK vs. M	0.952	0.001	0.002 **
CK vs. H	0.969	0.002	0.002 **
L vs. M	0.927	0.001	0.002 **
L vs. H	0.971	0.002	0.002 **
M vs. H	0.989	0.001	0.002 **

**Table 4:** Significance between soil physicochemical properties, enzyme activities and fungal community structure. Significance is denoted as follows: * *p* < 0.05, ** *p* < 0.01, *** *p* < 0.001.

Soil Factors	R^2^	*p*-Value
TN	0.7747	0.001 ***
pH	0.3099	0.012 *
BC	0.2629	0.021 *
SOC	0.2447	0.028 *
AP	0.8313	0.001 ***
WC	0.9424	0.001 ***
AN	0.9425	0.001 ***
AK	0.9593	0.001 ***
DOC	0.351	0.007 **
MBC	0.1242	0.183
FDA	0.961	0.001 ***
S-ACPT	0.9641	0.001 ***
S-DHA	0.803	0.001 ***
S-SC	0.5471	0.001 ***
S-UE	0.991	0.001 ***

## Data Availability

The original contributions presented in this study are included in the article/Supplementary Materials. The original data of this study are openly available in the NCBI SRA database with the sequence number: PRJNA1162305. Further inquiries can be directed to the corresponding authors.

## Supplementary Materials

The following supporting information can be downloaded at: https://www.mdpi.com/article/10.3390/jof11110772/s1, Figure S1: Heatmap of correlations between soil fungal genera, soil physicochemical properties, and enzyme activities Red identifies positive and blue identifies negative correlations, with darker colors for stronger correlations. Significance is denoted as follows: * *p* < 0.05, ** *p* < 0.01, *** *p* < 0.001.
