# Supplementary material for: High Fire Drives the Reorganization of Taiga Soil Fungal Communities with Ascomycota as the Dominant Phylum After Long-Term Recovery
Source: J Fungi (Basel). 2025 Oct 27;11(11):772. doi: 10.3390/jof11110772 (PMC12653922; doi:10.3390/jof11110772)
Supplement: Supplementary file 1 [file jof-11-00772-s001.zip › jof-3842946-supplementary.pdf]

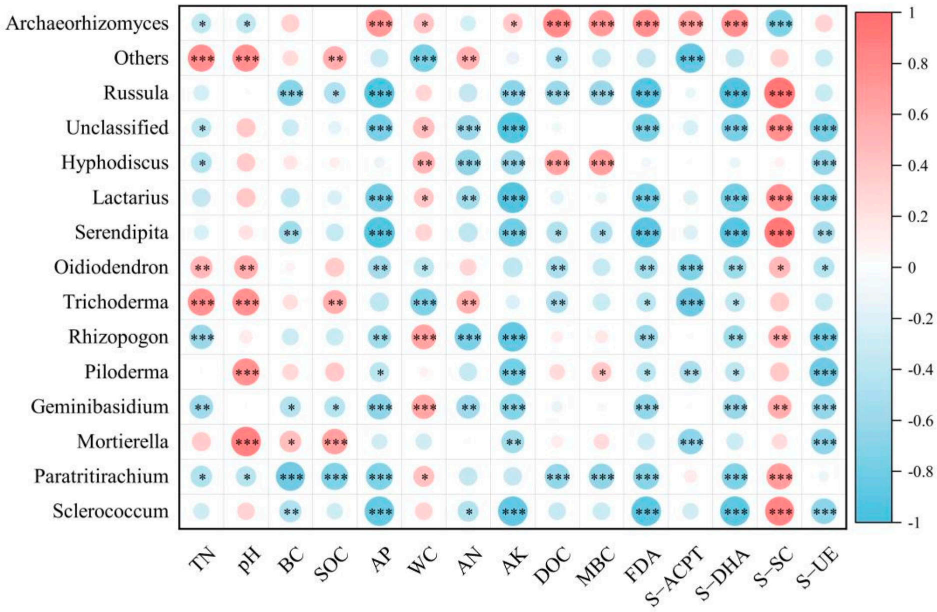

Figure S1. Heatmap of correlations between soil fungal genera, soil physicochemical properties, and enzyme activities Red identifies positive and blue identifies negative correlations, with darker colors for stronger correlations. Significance is indicated as * for $0.01 < p \leq 0.05$, ** for $0.001 < p \leq 0.01$, *** for $p \leq 0.001$.
